# Biogeography of *Rhaponticoides*, an Irano-Turanian element in the Mediterranean flora

**DOI:** 10.1038/s41598-022-24947-3

**Published:** 2022-12-20

**Authors:** Meryem Bozkurt, Juan Antonio Calleja Alarcón, Tuna Uysal, Nuria Garcia-Jacas, Kuddisi Ertuğrul, Alfonso Susanna

**Affiliations:** 1 Department of Biology, Faculty of Science, Selçuk University, 42130 Konya, Turkey; 2 Departament of Biology (Botany), Faculty of Sciences, Research Centre on Biodiversity and Global Change (CIBC-UAM), 28049 Madrid, Spain; 3 Botanic Institute of Barcelona (IBB, CSIC-Ajuntament de Barcelona), Pg. del Migdia, S.N., 08038 Barcelona, Spain

**Keywords:** Plant evolution, Phylogenetics, Speciation

## Abstract

Floristic relationships between the Irano-Turanian and Mediterranean regions have been known from old. However, only a few biogeographical analyses based on molecular data have evaluated the history of steppe plants within the Mediterranean basin. Our study aims to contribute to a better understanding of the migratory and diversification processes by reconstructing the biogeography of *Rhaponticoides* (Cardueae), distributed in the Mediterranean and Irano-Turanian regions. We generated nuclear and plastid sequences that were analyzed by Bayesian inference. We used the resulting phylogeny for dating the diversification of the genus and examining the dispersal pathways. Two clades were recovered, an Irano-Turanian clade and a Mediterranean clade. The origin of the genus was placed in the Anatolian plateau in the Middle Miocene. The genus experienced several diversifications and expansions correlated to the Messinian salinity crisis and the environmental changes in the Pliocene and the Quaternary. *Rhaponticoides* migrated following two routes reflecting the two souls of the genus: Irano-Turanian taxa colonized the steppes of Eurasia whilst Mediterranean species migrated via eastern and central Mediterranean and North Africa, leaving a trail of species; both pathways ended in the Iberian Peninsula. Our study also confirms that more work is needed to unravel phylogenetic relationships in *Rhaponticoides*.

## Introduction

The extraordinary biodiversity of the Mediterranean basin relays on its environmental heterogeneity, dramatic environmental fluctuations, millenial human influence, and a geographical position between temperate and subtropical regions, which makes it a tension zone^1–3^. However, the current floristic richness of the region has been also shaped by past climatic and geological events^4,5^. Inference from palaeobotanical remains and molecular data reveal that migration waves and speciation and extinction processes were driven by four main events: the cooling and aridification of the tropical climate during the Neogene; the Messinian salinity crisis; the onset of the Mediterranean climate; and the arid-cold fluctuations of the Quaternary^2,3,6^. Thus, the Mediterranean basin hosts a noticeable proportion of taxa from different biogeographic regions, and also a high number of endemics originated from extra-Mediterranean lineages^2,7,8^.

The steppe flora is one of the extra-Mediterranean elements that acquires special relevance within the Mediterranean basin^9,10^ contributing significantly to the richness of the Mediterranean flora^2,9^. It comprises taxa well adapted to continental climates with drastic temperature fluctuations between night and day and between different seasons (winter-summer) and high rainfall seasonality, usually in open habitats from plateaus and mountains^9–11^. The steppe flora exhibits a high diversity in central and western Asia, Arabian Peninsula, and North Africa^12,13^. Within this vast range, the Irano-Turanian region (central and western Asia) is the main core within the Holarctic kingdom that has exported numerous steppe taxa in multiple waves to adjacent regions throughout the Cenozoic^14,15^. The Irano-Turanian region shares with the Mediterranean basin numerous xerophilous plants that reach its westernmost extreme in the Iberian Peninsula, northern Morocco and southern Macaronesia^16^.

The intriguing presence of steppe flora in the Mediterranean basin has been recurrently explained by the Messinian Model^14^, which is primarily based on the Messinian salinity crisis that occurred at the Miocene-Pliocene boundaries^17^. Paleoenvironmental evidence supports the partial desiccation of the Mediterranean Sea and its ulterior restoration^14,17^. Both events would have fostered the east–west migration of the Asian steppe flora and the subsequent fragmented distribution of several taxa across the Mediterranenan basin and Asia^14,18^. This resultant geographical pattern is recognized as the “Kiermack disjunction”^19^. Some studies using fossil pollen and molecular data support a link between the Messinian salinity crisis (7–5 Ma) and the resultant disjunction pattern between the western and the eastern Mediterranean range, and western Asia^7,15,20^. In contrast, other studies show that recent climatic and sea-level oscillations throughout the Quaternary might have also facilitated the migration and the extant fragmentation of the steppe flora within the Mediterranean basin^18,21^. The Mediterranean basin was not covered by ice and permafrost like large portions of central and northern Europe during the glacial periods, but it did experience climates similar to those of the current steppes^22^. Likewise, in these cold periods the sea level fell by nearly 130 m, favoring the emergence of lands within the Mediterranean Sea^23,24^. Thus, suitable climates and land bridges could have fostered the westward expansion of the steppe flora from Asia throughout the Quaternary^18,21^.

The colonization of the Mediterranean basin from Asia might have occurred by different routes^14,25,26^. Taxa from cold Asian steppes would have expanded through the northern arc of the Mediterranean basin and central Europe, colonizing open habitats such as the Pannonian region and subalpine environments^11,27^. On the other hand, thermophilous taxa (e.g. from the Arabian Peninsula) would have migrated through the northern rim of Africa^26,28^. In parallel to migrations and expansions or contractions of the ranges of the steppe flora^11^, speciation processes have taken place^3,8,28,29^. Several taxa with an Asian origin diversified in steppe but also non-steppe environments in their westward expansion across the Mediterranean basin throughout late Cenozoic^15,28,30^.

One of the most characteristic elements of the Irano-Turanian and Mediterranean flora are the thistles (Compositae tribe Cardueae; cf.^31,32^. Cardueae are one of the largest tribes of the family with about 2500 species^33,34^ and most of its subtribes and some very speciose genera such as *Centaurea*, *Cirsium*, *Cousinia*, *Jurinea,* and *Saussurea* exhibit diversity centers in Asia^35–38^. Recent dating analyses have revealed that they originated in west Asia with expansions and radiations to the Mediterranean and Middle Asia throughout the Cenozoic^37^. The phylogeny and distribution of tribe Cardueae have been thoroughly studied, but such a large tribe offers multiple examples that are worth delving into and one of them is the genus *Rhaponticoides* (subtribe Centaureinae).

**Figure 1:**
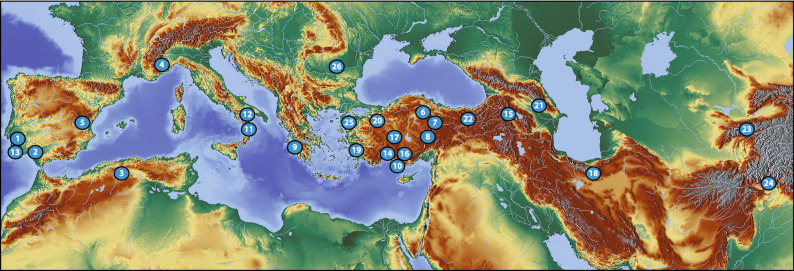
Geographical distribution of the genus and our sampling. The number for each population refers to Table 1. The figure was created by using http://maps-for-free.com/ and Adobe Illustrator_26.5.

According to the latest revision, *Rhaponticoides* comprises 36 species, either narrow endemic or widespread taxa ranging from the steppes of Mongolia to the Iberian Peninsula and North Africa^33,39–41^; Fig. 1. This figure, however, should be taken with caution because it is surely an overestimation (see below Sampling in Matherial and Methods for details). From a systematic point of view, *Rhaponticoides* is considered an orphan genus in the Centaureinae because it is especially isolated within the subtribe and we ignore the identity of any potential relative or sister genus^35,37^. Despite its biogeographic interest^42^, we lack a time-calibrated biogeographical study to shed light on the processes underlying the current distribution of the genus and reveal its ancestral area as well as its migration routes and speciation events across the Mediterranean basin. Similarly, a comprehensive phylogenetic study based on molecular data of the genus might support or refute the latest taxonomical proposal^41^ as well as previous morphological, palynological, and karyological studies^39,40,43–46^.

The biogeographical studies based on molecular data of some floristic elements with remarkable disjunctions within the Mediterranean basin and nearby territories, such as the Arctic Alpine Flora or the Rand Flora, have revealed that the observed chorological patterns are shaped by much more complex processes than those provided by classical historical biogeography^7,47–49^. The observed distribution and speciation patterns of the steppe within the Mediterranean basin have been, comparatively, much less studied and usually resolved by invoking the Messinian model^14,19^. To date, few modern biogeographical studies have tested explicitly or implicitly the Messinian Model. They usually provide scenarios with events and processes within different time frameworks according to the taxa studied^15,28,50^.

In this context, the present study aims to contribute to a better understanding of migratory and diversification processes underlying the current chorological patterns of taxa related to the Asian steppe flora. We used two nuclear regions (ITS and ETS) and two plastid regions (*rpl32-trnL*^*UAG*^ and *ycf3-trnS*) and carried out Bayesian inference, dating, ancestral area reconstruction, and dispersal analyses with the following specific objectives: (1) to elucidate the phylogenetic relationships of the *Rhaponticoides* species; (2) to reconstruct the biogeographical history using dating and ancestral area reconstruction analyses and test if the genus truly has a geographical origin linked to the Asian steppes; (3) to redraw the expansion of *Rhaponticoides* through the Eurasian steppes and the Mediterranean basin and reconstruct both the pathway and the drivers of its expansion through Eurasia and North Africa.

## Results

### Phylogeny of *Rhaponticoides*

**Figure 2:**
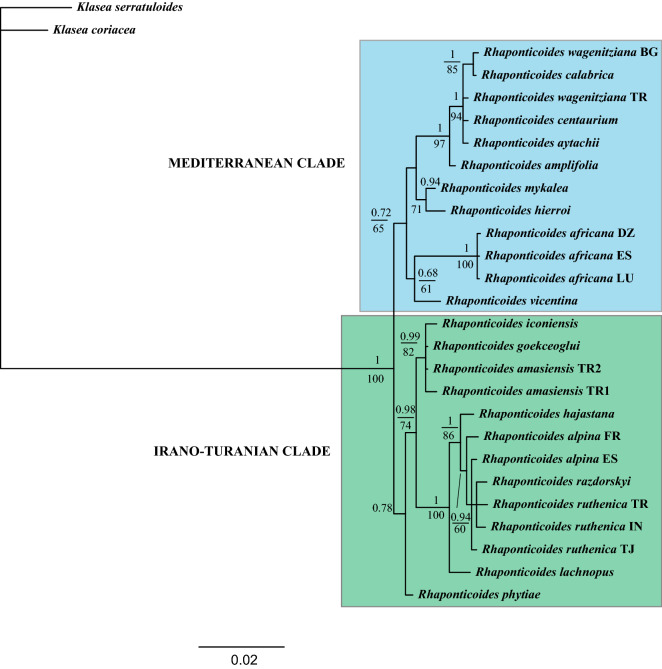
50% majority-rule consensus tree obtained by Bayesian analysis of the combined nuclear dataset, indicating supported clades. Numbers occurring above branches are posterior probabilities (PP lower than 0.6 are not shown). Likelihood Bootstrap values figure below branches (BS lower than 60% are not shown). Capital letters following the names of species correspond to the countries of origin for species with more than one sample (see Table 1). *BG* Bulgaria, *DZ* Algeria, *ES* Spain, *FR* France, *IN* India, *PT* Portugal, *TJ* Tajikistan, *TR* Turkey.

Species of Rhaponticoides are placed in the nuclear phylogeny in two moderately-supported clades that coincide with the dual distribution of the genus (Fig. 2). The most speciose one is the Irano-Turanian clade, which encompasses all the representatives from the Irano-Turanian part of Turkey, Armenia, and Iran, plus the species from the steppes of Eurasia. The Mediterranean clade comprises taxa from the Mediterranean part of Anatolia, south Balkans, and the Italic and Iberian peninsulas.

**Figure 3:**
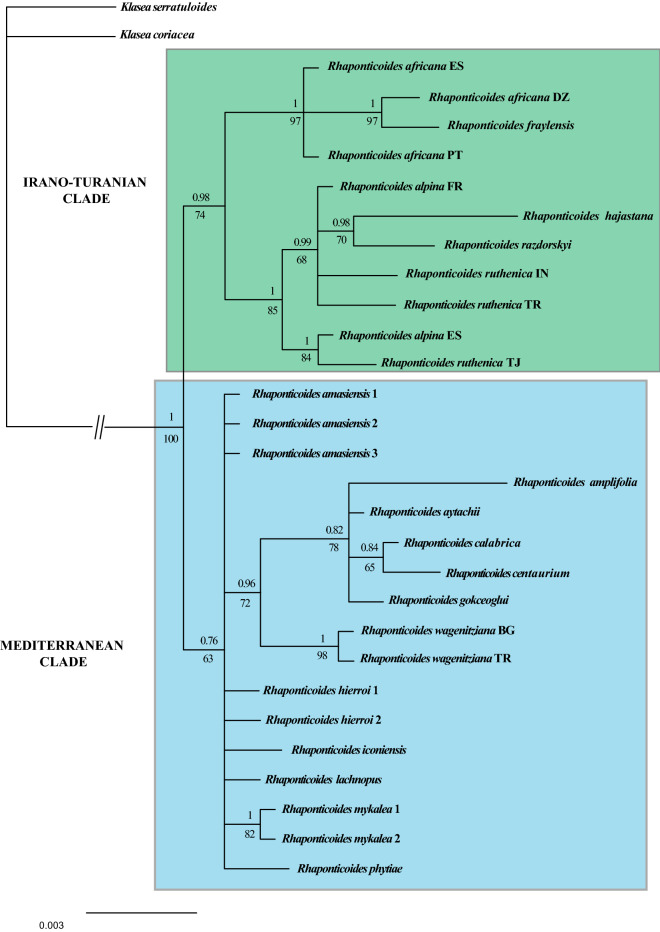
50% majority-rule consensus tree obtained by Bayesian analysis of the combined plastid dataset, indicating supported clades. Numbers occurring above branches are posterior probabilities (PP lower than 0.6 are not shown). Likelihood Bootstrap values figure below branches (BS lower than 60% are not shown). Capital letters following the names of species correspond to the countries of origin for species with more than one sample (see Table 1). *BG* Bulgaria, *DZ* Algeria, *ES* Spain, *FR* France, *IN* India, *PT* Portugal, *TJ* Tajikistan, *TR* Turkey.

Both the nuclear (Fig. 2) and plastid DNA (Fig. 3) phylogrames are largely coincident, with a few hard incongruences: *Rhaponticoides africana* and *Rh. fraylensis* are placed within the Mediterranean clade by the nuclear data (Fig. 2) and in the Irano-Turanian clade in the plastid phylogeny (Fig. 3). Similarly, *Rh. iconiensis*, *Rh. gokceoglui*, *Rh. amasiensis* and *Rh. phytiae* are placed in the Irano-Turanian Clade in the nuclear phylogeny (Fig. 2) whereas the same four species are located in the Mediterranean Clade in the plastid tree, albeit only the position of *Rh. gokceoglui* is supported (Fig. 3). In any case, the very low resolution of the plastid phylogeny results in a virtually unsupported Mediterranean Clade.

### Biogeography within a time-calibrated framework

**Figure 4:**
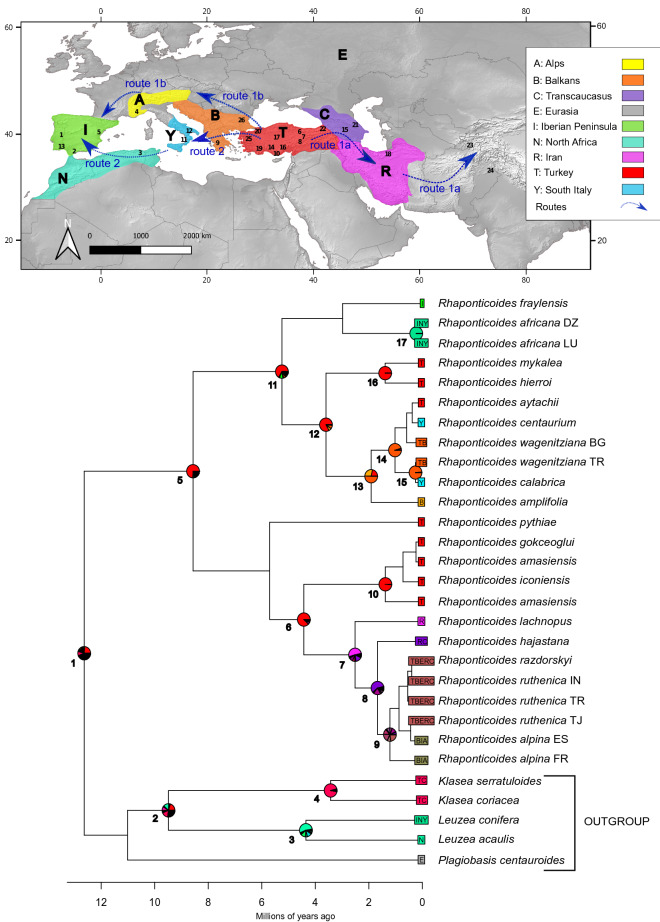
Molecular dating and biogeographic analyses. Maximum clade credibility tree from the relaxed molecular-clock analysis with exponential distribution and Birth and Death speciation process of ITS and ETS sequences in BEAST. Numbers refer to the supported nodes (Table 3). Pie charts (shown only for supported nodes, Table 3) reflect the relative probability of each area or combination of areas being ancestral, according to the ancestral area reconstructions based on the BayArea + J model implemented in BioGeoBEARS. Letters correspond to the ancestral areas or combination of areas represented in the upper part of the figure. The figure was created by using http://maps-for-free.com/, QGIS_3.4, and Inkscape_0.92.3.

The BayArea + J model performed best to reconstruct the ancestral area of *Rhaponticoides* (Table S2). The final time-calibrated biogeographic model revealed that Turkey is the most plausible ancestral area of *Rhaponticoides* in the middle of the Miocene (12 Ma). The genus diverged in two geographical lines, the Irano-Turanian and Mediterranean clades, in the Late Miocene (8 Ma). Both branches diversified again in the Miocene-Pliocene transition (5 Ma) and show a radiation process in the Pliocene–Quaternary transition and throughout the Quaternary period (Fig. 4, Table 3).

The diversification processes entailed different expansion processes towards western and central Asia, on the one hand, and towards the Mediterranean basin on the other. This diversification occurred in different lineages and on different dates. The Irano-Turanian steppe line originated in the steppe environments of Turkey, i.e., the Anatolian plateau (red region in Fig. 4) and diversified (e.g. *Rh. amasiensis*) (nodes 6 and 10 in Fig. 4). This line also expanded towards Iran and the Caucasus (nodes 7 and 8, route 1a in Fig. 4, Table 3), and gave rise to the Iranian species complex of taxa (*Rh. lachnopus*) and the Caucasian group (*Rh. hajastana*). The same branch also originated the *Rh. alpina/ruthenica* complex, which integrates populations that occur from West India through the Alps and Mediterranean basin up to the Iberian Peninsula. These European populations (yellow and green areas in Fig. 4) represent a westward migration track (route 1b in Fig. 4) across the northern rim of the Mediterranean basin.

The Mediterranean line (node 11 in Fig. 4) also exhibits an Asian origin with ulterior diversification of species. This line shares with the steppe one the ancestral area in Turkey (red area in Fig. 4), and originated a first clade (nodes 11 and 12 in Fig. 4) in southern Anatolia associated with the Mediterranean climate (*Rh. hierroi* and *Rh. mykalea*). This Mediterranean line reached the Balkans (*Rh. amplifolia* and *Rh. wagenitziana*) and south Italy (*Rh. calabrica* and *Rh. centaurium*) constituting a Balkan-Greek-Italic lineage (nodes 13, 14, and 16, route 2 in Fig. 4). The Mediterranean line also expanded across the Mediterranean habitats from south Italy (*Rh. africana*) to North Africa (*Rh. africana*, *Rh. eriosiphon* [presumibly extinct, Susanna pers. comm.]) up to the Iberian Peninsula (*Rh. africana* and *Rh. fraylensis*) (node 17, route 2 in Fig. 4).

## Discussion

*Origin of* Rhaponticoides*, tempo, and location.* Steppe flora and vegetation are present in the Mediterranean basin (comprised Western Europe) since the Paleogene and gained relevance throughout the Neogene, especially during the Miocene^10,20^. The geographical origin of the main steppe lineages that colonized the Mediterranean basin is Central and Western Asia^15,30,51^ and this is also the case for *Rhaponticoides* (Fig. 4) because Turkey appears as the most probable ancestral area. Turkey's landscape is dominated by the Anatolian plateau, which belongs to the Irano-Turanian region^15,52^, and a Mediterranean belt located in the south and west of the Anatolian Peninsula.

The Anatolian plateau, located at the intersection of Europe, Asia, and Africa, benefits from the diversity of three hotspots: East Mediterranean, Iran-Anatolia, and the Caucasus^29,50^. It is also a meeting place and dispersal corridor for different lineages that originated in Asia and colonized Europe and North Africa during the Cenozoic^15,53^, among them subtribe Centaureinae to which *Rhaponticoides* belongs^37^. Moreover, Anatolian plateau exhibits a high level of endemism and rich biodiversity probably driven by a complex paleogeographic history with dramatic topography and climate changes during the Cenozoic^54,55^.

The origin of *Rhaponticoides* should be placed in Middle Miocene as suggested for different taxa of vascular plants of Irano-Turanian origin^50,56^ and many groups from the Compositae^33,34^. Most diversification events within Cardueae and especially in subtribe Centaureinae are related to recurrent connection and isolation episodes between Anatolian plateau and the Mediterranean basin throughout the Miocene^37^. In turn, these episodes are linked to environmental changes across the Irano-Turanian and Mediterranean regions such as climatic changes, normally tending to cooling and aridification, and collisions of tectonic plates and subsequent uplifts of plateaus and mountains comprising those close to Anatolia, e.g., Zagros^29,50^.

*Diversification of* Rhaponticoides*.* The diversification of *Rhaponticoides* took place in the late Cenozoic (Fig. 4) as described for other vascular plant lineages^57^. It started at the end of the Miocene with subsequent radiation events in the Miocene-Pliocene and Pliocene–Quaternary transitions (Fig. 4). The first diversification events in the late Miocene and the Miocene-Pliocene transition especially implied dispersion. As registered in other steppe Irano-Turanian taxa^25,49^, *Rhaponticoides* steppe lineage expanded eastwards and originated species currently growing in Iran like *Rh. lachnopus*. In parallel, the genus shows a Mediterranean line that originated in the Mediterranean region of Turkey, which migrated to the west reaching southwestern Europe and northwestern Africa. These dispersal and diversification events coincide with those inferred in other steppe xerophytes that are present in the Mediterranean basin such as *Anabasis*^28^, *Acantholimon*^29^, and *Haplophyllum*^15^. Expansion of Irano-Turanian xerophytes had been favored by the extreme aridification and partial desiccation of the Mediterranean Sea during the Messinian crisis, which would have led to landmass connections, i.e., corridors towards the western end of the Mediterranean basin. Thus, the early diversification of *Rhaponticoides* fits the Messinian Model^14^.

The Messinian Model is compatible in turn with posterior dispersal and diversification processes that occurred during the Pliocene–Quaternary transition and Quaternary that may be related to Mediterranean climate onset^8,58^ and also associated with the climatic and sea-level oscillations associated with glaciations^21,28,29^. The main lineages of *Rhaponticoides* which were previously originated in the Miocene-Pliocene transition experienced diversification and colonized the Mediterranean basin and western Asia. The Mediterranean line evolved probably from the steppe line into species that occurred in the Mediterranean belt of Anatolia (e.g. *Rh. amasiensis*, *Rh. hierroi*, *Rh. mykalea*) and in Armenia (*Rh. hajastana*), and then migrated to the eastern and central Mediterranean (Balkans and Italy, e.g., *Rh. amplifolia, Rh. calabrica*, *Rh. centaurium*, *Rh. wagenitziana*). In addition, within the steppe line, some species emerged and achieved wide and disjunct distributions either by fragmentation of a wide original range or by long-distance dispersal events^59^. This is the case of the *Rh. alpina/ruthenica* complex with disjunct populations in different mountains: from the Tian Shan in central Asia to the Caucasus, Balkans, European Alps and the Iberian Peninsula^41,42^. Similar results have been obtained in other steppe taxa originated in the Irano-Turanian Region^28,29,51^.

Considering the environmental variations throughout the late Cenozoic, vicariance could explain, at least partially, the origin of lineages and species of *Rhaponticoides*. However, speciation by dispersal events seems to be a lot more frequent than vicariance in steppe taxa located in the Mediterranean and Irano-Turanian regions^15,29,30,51^. In our analyses, BayArea + J was selected as the best model, and this model embraces cladogenesis and sympatry as well as anagenesis and dispersion whereas vicariance has no relevance^60^. *Rhaponticoides* species of the steppe and Mediterranean lines would have originated in regions with biogeographical particularities and complex environmental history such as the Anatolian plateau, which harbors multiple endemic species^54,55^. Range expansions were favored by the corridors enabled by events such as the Messinian crisis^15,29^.

The incongruence between plastid and nuclear data detected in Iberian *Rh. fraylensis* and *Rh. africana* (Figs. 2 and 3) is very illustrative on the origin of the Mediterranean line within the Irano-Turanian pool: both species appear in the plastid phylogeny nested in the Irano-Turanian clade. The usual explanation for these inconsistencies is ancient hybridization and subsequent plastid capture^61,62^. In our case, the most plausible hypothesis suggests that the ancestor of *Rh. africana* and *Rh. fraylensis* acquired the Irano-Turanian chloroplast by introgression in the contact zone between both clades in Anatolia. The other incongruences between plastid and nuclear phylogenies (i.e., *Rh. amasiensis*, *Rh gokceoglui*, *Rh. iconiensis* and *Rh. phytiae*) can be reduced to only one supported incongruence in *Rh. gokceoglui* since the position of *Rh. amasiensis*, *Rh. iconiensis* and *Rh. phytiae* is unsupported in the plastid tree (PP = 0.76, BS = 63%, Fig. 3). The conflicting position of *Rh. gokceoglui* should be also explained by an ancient event of hybridisation and plastid capture, which is a common fenomenon in tribe Cardueae^33,36^.

*Migration routes toward the Mediterranean basin.* To date, two routes have been usually reported for the Irano-Turanian steppe flora currently located within the Mediterranenan basin. Some taxa followed a “northern route” encompassing mountains and/or steppes of southern Europe and the northern rim of the Mediterranean Sea^11,27^. In contrast, other taxa tracked a “southern route” through North Africa, the Mediterranean islands and eventually through landmasses that emerged during the arid crises of the late Neogene and Quaternary^26,28^. *Rhaponticoides* comprises lineages that match both routes and reflect both biogeographic patterns (routes 1b and 2 in Fig. 4).

Regarding the north route (route 1b in Fig. 4), the steppe line that originated the *Rh. alpina/ruthenica* complex reached the western edge of the Mediterranean basin (Iberian Peninsula) through the Balkans and Alps, leaving in both massifs relictic populations of *Rh. alpina* as milestones^42^. This line also encompasses species from the western and central Asian steppes with a distribution centered on the Irano-Turanian region reaching the steppes of Iran in the south (represented in our analyses by *Rh. lachnopus*) (Fig. 4). As for the south route (route 2 in Fig. 4), the Mediterranean line integrates a series of species of narrow distribution, many of them from the Mediterranean part of Anatolia^41,46^ extending westwards to North Africa and the Iberian Peninsula (Fig. 4).

The coexistence of both pathways and the arrival of the two lines—steppe and Mediterranean—to the Iberian Peninsula matches the case of *Centaurea* sect. *Acrocentron* (Cass.) DC.^25^. The pathway of *Centaurea* from Sicily to Spain is punctuated by some relict species that stand as stepping-stones, namely (from east to west) *C. tauromenitana*, *C. carolipauana*, and *C. clementei*. In contrast, stepping-stones in the south pathway of *Rhaponticoides* consist only in relict, isolated populations of a single species, namely *Rhaponticoides africana*, with small stands in Sicily, North Africa, and south and NW Iberia. The remarkable journey of *Rh. africana* ended in Galicia (north-west Spain), where some relict populations barely survive on the coastal scree^63^.

*Taxonomic implications*. The aims of this paper are not taxonomic, but our results show that the taxonomy of the genus is far from being well-known. The infrageneric taxonomy of *Rhaponticoides* has been studied for long but primarily based on morphological criteria. The most exhaustive and recent work^41^ proposed a sectional classification of the genus in which virtually every species is a section or subsection, much in agreement with^39^. However, our results reveal discrepancies in the monophyly of some sections and species. Sections *Ruthenicae* and *Aralocaspicae* are not monophyletic and both of them include species recovered into different and unrelated clades. Such inconsistencies might arise from the species delimitation made by^39^. For instance, the morphological evidence for separating *Rh. ruthenica* from *Rh. alpina* are extremely weak and merely based on some vegetative characters^41^ whereas our phylogenetic results confirm that this segregation lacks support from molecular evidence (Fig. 2).

In the same line, many taxonomic entities at species level of quite doubtful value in our opinion were described within the wide range *Rh. ruthenica*. Supporting this view, our results place *Rh. razdorskyi*, one of these segregate species, grouped with *Rh. ruthenica*. Within the same section *Ruthenicae*, the independence of *Rh. linaresii*, represented in our analyses by the Valencia population of *Rh. alpina* and sustained by^41^, has been rejected by all revisions of the group^42,64^, who merged it into *Rh. alpina* as confirmed by our results. Similarly, *Rh. carrisoi* is listed as separate species in^41^ but it is actually a synonym of *Rh. fraylensis*^64^. Finally, *Rh. centaurium* and *Rh. calabrica* have been considered synonyms^65^ which, according to our results, is highly probable. Instead, the species would be very narrowly connected to *Rh. wagenitziana* from the Balkans^45^.

In sum, the global richness and delimitation of some infrageneric entities, sections, and species in *Rhaponticoides* requires a deep, serious revision that should be carried out within the framework of the integrative taxonomy^66^. We suggest incorporating a wider representation of taxa and the use of the phylogenomic approach already used in the Cardueae^37^.

## Concluding remarks

The biogeographical history of the genus set its origin in the Irano-Turanian part of Turkey (Anatolian plateau) in the Middle Miocene. The genus experienced different diversifications and westward and eastward expansions related not only to the Messinian salinity crisis but also to ulterior environmental changes in the Pliocene–Quaternary and Quaternary periods. *Rhaponticoides*, like other Irano-turanian steppe taxa, colonized the Mediterranean basin on different dates. However, in contrast to most of the previously studied taxa, the genus *Rhaponticoides* migrated and diversified across the Mediterranean basin following two different routes. The construction of the phylogenetic relationships within *Rhaponticoides* reveals the urgent necessity of a comprehensive integrative study on the genus to resolve the delimitation of some infrageneric taxa.

## Material and methods

The methods comply with local and national guidelines.

### Sampling

Sampling was designed to cover all the area and the nuclei of diversification of the genus: Caucasus, Iran, Balkans, Italian and Iberian peninsulas, Sicily, North Africa, and the steppes of Eurasia, reaching the extreme of the area in India (Fig. 1). Special focus was posed in Turkey, where the genus shows its peak of species according to^44^. Most of the species listed by^39,41^ that are missing from our sampling are doubtful segregates from the widespread *Rh. ruthenica*; in fact, the total number of species of the genus is probably closer to 25–30, which makes our coverage 60–72% of the genus. In view of the differences in the treatment of some taxa and the very large area of some others, we included several populations of *Rhaponticoides africana*, *Rh. alpina* and *Rh. ruthenica*. Sampling totals 26 populations of 18 species. For the phylogenetic analyses, outgroup was chosen following^37^ and included two species of genus *Klasea* Cass., namely *Klasea coriacea* (Fisch. & C.A. Mey. ex DC.) Holub and *K. serratuloides* (DC.) Greuter & Wagenitz (datasets 1 and 2). For the dating analyses, other outgroups were added (see below, dataset 3). The origin of the materials and vouchers are indicated in Table 1.

**Table 1:** Code represented in Fig. 1 and description of the geographical origin of samples of *Rhaponticoides* (*Rh.*) species and their GenBank accession numbers of the sequences included in phylogenetic and biogeographical analyses.

Map code	Species and population	Locality	GenBank Accesions			
			ITS	ETS	*ycf3-trnS*	*rpl32-trnL*
1	*Rh. africana PT*	PORTUGAL: Serra da Arrábida, crossroad Monastir—Portinho da Arrabida, 38°27′40.1ʺN 9° 00′38.3ʺW, 254 m, *Garcia-Jacas & Vilatersana 2355* (BC)	ON694085	ON745226	ON745250	ON745276
2	*Rh. africana ES*	SPAIN, Cádiz: Chiclana, Cabo Roche, near “Venta El Colorao”, *Balao, Ortiz & Talavera s/n,* 15-5-2008 (SEV224830)	ON694086	ON745227	ON745251	ON745277
3	*Rh. africana DZ*	ALGERIA: above Tigzirt (N of Tizi Ouzou), 50 m, sandstone slopes, *Davis 5917* (E00286681)	ON694087	ON745228	ON745252	ON745278
4	*Rh. alpina FR*	FRANCE, Alpes-Maritimes: Sospel, Vallon de Saint Julien, *Huynh-Tan 274* (HYE)	ON694088	ON745229	ON745253	ON745279
5	*Rh. alpina ES*	SPAIN, Valencia: Teresa de Cofrentes (Ayora-Cofrentes valley), 1000 m, *Riera & Ferrer-Gallego s/n*, 22-06-2010 (VAL202212, voucher; *Ferrer-Gallego s/n,* 19-6-2016, leaves)	ON694089	ON745230	ON745254	ON745280
6	*Rh. amasiensis TR1*	TURKEY, Amasya: Abaci mountain, 920 m, *Demirelma 3287 & Uysal* (KNYA)	ON694090	ON745231	ON745255	ON745281
7	*Rh. amasiensis TR2*	TURKEY, Amasya: Abaci mountain, Abaci village entrance, 402,417 N, 353,554 E, *Juniper* forest openings to the right of the road, 675 m, *Demirelma 3280 & Uysal* (KNYA)	ON694091	ON745232	ON745256	ON745282
8	*Rh. amasiensis TR3*	TURKEY, Yozgat: Akdağmadeni, deciduous forest, N 4391835.080 E 723741.880, 1357 m; and N 4391864 E 723722.658 1352 m, *Ekizi* (GAZI)	––	––	ON745257	ON745283
9	*Rh. amplifolia*	GREECE: Mount Killini, *Constantinidis 12,434 & Vassiliadis* (ATHU)	ON694092	ON745233	––	ON745284
10	*Rh. aytachii*	TURKEY, Karaman; Sarıveliler, Göktepe road, Dumlugöze, forest openings, 1330 m, 19.06.2017, *Bozkurt, Ertuğrul 5359 and Uysal* (KNYA)	ON694093	ON745234	ON745258	ON745285
11	*Rh. calabrica*	ITALY, Cosenza: Saracena, *Rosati s/n*, 20-Jun-2015 (HLUC)	ON694094	ON745235	ON745259	ON745286
12	*Rh. centaurium*	ITALY, Matera: near Salandra, *Rosati s/n*, s/d (HLUC)	ON694095	ON745236	ON745260	ON745287
13	*Rh. fraylensis*	PORTUGAL, Beja: Vilanova de Milfontes, road M532 to Pinheiro, 37° 43.210′N, 8° 42.908′W, banks and ditches of the road, 81 m, *Garcia Jacas & Susanna 2801* (Susanna pers. herb.)	ON694096	ON745237	ON745261	ON745288
14	*Rh. gokceoglui*	TURKEY, Antalya: İbradı, ascent to Melik Yayla above Başlar Village, Suçıkan, *Pinus nigra* forest openings, limestone, *Bozkurt & Ertuğrul 5611* (KNYA)	ON694097	ON745238	ON745262	ON745289
15	*Rh. hajastana*	ARMENIA, Shirak: between Pokr Arthik and Bagravan, *Susanna 1587 *et al*.* (BC)	AY826235	DQ310959	MK598510	JF754895
16	*Rh. hierroi TR1* and *TR2*	TURKEY, Antalya: Saklıkent road, after Yazır village, 1150 m, *Ertuğrul 4153a* (KNYA)	ON694098	ON745239	ON745263 ON745264	ON745290 ON745291
17	*Rh. iconiensis*	TURKEY, Konya: between Bozkır and Seydişehir, field margins, 1170 m, *Ertuğrul, Tugay 4932 & Vural* (KNYA)	ON694099	ON745240	ON745265	ON745292
18	*Rh. lachnopus*	IRAN, Semnan: 20 km N of Semnan, 1500 m, *Susanna 1639 *et al*.* (BC)	ON694100	ON745241	ON745266	ON745293
19	*Rh. mykalea TR1* and *TR2*	TURKEY, Aydın; Kuşadası, Davutlar road, landside, 50 m, *Ertuğrul 4765a* (KNYA)	ON694101	ON745242	ON745267 ON745268	ON745294 ON745295
20	*Rh. phytiae*	TURKEY, İzmit; Karamürsel, between Suludere and Safiye, entrance of Safiye village*, Sağıroğlu 3965* (SAKU)	ON694102	ON745243	ON745269	ON745296
21	*Rh. razdorskyi*	AZERBAİJAN: near Shemakhi [Samaxi], *Tzevelev & Czerepanov s/n*, 12-Jun-1957 (E00474025)	ON694103	ON745244	ON745270	ON745297
22	*Rh. ruthenica TR*	TURKEY, Muş-Malazgirt: way to Karıncalı village 5 km from Kazgöl, 1760 m, clay slopes, *Ertuğrul 4501 & Uysal* (KNYA)	ON694104	ON745245	ON745271	ON745298
23	*Rh. ruthenica TJ*	TAJİKİSTAN: Maijora canyon on the road to the mines, N39° 03′ 44.8ʺ, E68° 43′ 15.5ʺ, *Susanna 2471 *et al*.* (Susanna pers. herb.)	ON694105	ON745246	ON745272	ON745299
24	*Rh. ruthenica IN*	INDİA: Siru Gol S of Shan Jinali Pass, 11000ft, dry slopes*, J.D.A. Stainton s.n.,* 02-Aug-1958 (E00286756)	ON694106	ON745247	ON745273	ON745300
25	*Rh. wagenitziana TR*	TURKEY, Istanbul: Kartal, Aydos mountain, 370 m, *Uysal 3748* (KNYA)	ON694107	ON745248	ON745274	ON745301
26	*Rh. wagenitziana BG*	BULGARİA, Yambol: Toudzha Hills, Municipality of Elhovo, north of Golam Dervent, 348 m, 42°01′N, 26°43′E, *Bancheva & Stoyanov s/n*, 26-7-2006 (SOM163690)	ON694108	ON745249	ON745275	ON745302
OUTG	*Klasea coriacea*	ARMENIA, Ararat: pass between Tigranashen and Sovetashen, 2200 m, *Susanna 1538 *et al*.* (BC)	DQ310926	DQ310965	MK598512	JF754881
	*Klasea serratuloides*	ARMENIA, Kotayk: between Gherart and Garni, *Susanna 1569 *et al*.* (BC)	AY826295	DQ310962	–-	JF754882
	*Leuzea acaulis*	ALGERIA, Laghouat: 16 km north of Aflou, *J. M. Montserrat 2334 *et al*.* (BC)	AY826334	DQ310995	–-	–-
	*Leuzea conifera*	SPAIN, Girona: sine loc., *Font s/n*, 7-1993 (BC)	AY826298	DQ310996	–-	–-
	*Plagiobasis centauroides*	KAZAKHSTAN, Almatynski ob.: Narienkul road 2 km to the Charin river bridge, *Susanna 2130 *et al*.* (BC)	AY826312	KU324157	–-	–-

*BG* Bulgaria, *DZ* Algeria, *ES* Spain, *FR* France, *IN* India, *PT* Portugal, *TJ* Tajikistan, *TR* Turkey.

### DNA extraction and amplification

Total genomic DNA was extracted from silica gel-dried tissue of one plant per population. The extraction of DNA followed the CTAB method of^67^ with the modifications of^68^ including three washing steps with sorbitol buffer. The ITS, ETS, *rpl32-trnL*^*UAG*^*,* and *ycf3-trnS* regions were amplified by polymerase chain reaction (PCR). The amplification primers for the nuclear regions were ITS1 and ITS4^69^ for the ITS, and ETS1F^70^ and 18SETS ^71^ for the ETS region. For the plastid *rpl32-trnL*^*UAG*^ region, we used rpl32-F as the forward primer and trnL^UAG^ as the reverse primer^72^. For the plastid *ycf3-trnS* region, we used SP43122F as the forward primer and SP44097R as the reverse primer^73^. The PCR reactions were performed under the conditions detailed in^74^. The amplified DNA segments were sequenced on an ABI 3730XL Analyser (Applied Biosystems, Foster City, CA, USA) following the manufacturer’s protocol at Macrogen Inc., Korea.

### Phylogenetic analyses

Nucleotide sequences were edited using Bioedit v7.0.5.3^75^ and aligned visually by sequential pairwise comparison^76^. Basic data on ITS, ETS, *rpl32-trnL*^*UAG*^*,* and *ycf3-trnS* matrices are available in Table S3. The ITS plus ETS matrix was 1594 bp long (dataset 1) and the aligned plastid matrix was 1821 bp long (dataset 2). Likelihood analysis of both datasets was carried out by heuristic search using PAUP v4.0^77^ using TBR branch swapping with character states specified as unordered and unweighted. The likelihood criterion was set to HKY85 (the default option in PAUP). Bootstrap analyses^78^ were performed using 100 replicates of heuristic search with the default options. Internodes with a Bootstrap (BS) value > 75% were considered statistically significant.

Bayesian inference of the two datasets was calculated using MrBayes v3.2.6^79^. The best available model of molecular evolution required for Bayesian estimations of phylogeny was selected using Akaike information criteria (AIC) for both datasets as implemented in the software MrModeltest v2.2^80^. The best fitting model was GTR + G + I for both datasets. Bayesian inference analyses were initiated with random starting trees and were run for 40 × 10^6^ generations in two independent runs of four Metropolis-coupled chains. We saved one out of every 1000 generations, resulting in 40,000 sampled trees. Data from the first 1000 sampled trees were discarded as the ‘‘burn-in’’ period, after confirming that log-likelihood values had stabilized prior to the 1000th sampled tree. The stationarity of the runs and the convergence between the runs were checked with Tracer v.1.5.0^81^. Internodes with posterior probabilities (PP) > 0.95 were considered statistically significant.

### Dating analyses

Divergence times were estimated using the nrDNA (ETS and ITS) sequences organized in a matrix with one-two accessions for each taxon of the ingroup (dataset 3, Table 1). The outgroups included *Klasea coriacea*, *K. serratuloides* (as in dataset 1) plus *Plagiobasis centauroides* Schrenk, *Leuzea acaulis* (L.) Holub., and *L. conifera* (L.) DC. Dating analyses were performed by using BEAST v.1.8.4. Four monophyletic groups were defined in BEAUti v.1.8.4 (included in BEAST package): (i) all species of dataset 2, (ii) *Plagiobasis centauroides*, (iii) *Klasea* plus *Leuzea* clade, (iv) and *Rhaponticoides* genus. These four groups were also implemented as secondary calibration points based on a previous phylogenomic study focused on the Cardueae tribe^37^, see Table 2.

**Table 2:** Summary of the calibration points and the prior distribution applied in dating BEAST analyses represented in Fig. 4.

Calibration point	Median age Ma (95% HPD interval Ma)	Prior distribution	Node	Source
1	12.39 (11.39–13.66)	Normal	Root of the tree (node 1)	Herrando-Moraira et al*.* (2019) (node 57)
2	11.42 (10.52–12.55)	Normal	Stem *Plagiobasis centauroides*	Herrando-Moraira et al*.* (2019) (node 58)
3	9.55 (8.7–10.58)	Normal	*Klassea* & *Leuzea* (node 2)	Herrando-Moraira et al*.* (2019) (node 61)
4	8.74 (7.82–9.64)	Normal	*Rhaponticoides* (node 5)	Herrando-Moraira et al*.* (2019) (node 71)

To obtain the best time-calibrated phylogram, models with strict and uncorrelated log-normal relaxed clocks were run under two different speciation tree models, Yule and birth–death^82,83^. The options of lognormal and exponential distributions were also tested in the case of models with log-normal relaxed clocks (see Table S1). The six resulting BEAST models were run for four independent chains of 50 million generations each, sampling every 1000 generations. Their convergence was assessed by confirming that all parameters had reached stationarity and sufficient effective sample sizes (> 200) in all converged runs using Tracer v1.7^81^. All models and replicates were run in CIPRES Science Gateway^84^.

**Table 3:** Age estimates and reconstructed ancestral ranges for each of the nodes in the chronogram represented in Fig. 4.

Node	BPP	Interval age–HPD 95%	BayArea + J model probabilities
1	1	11.73–13.54	T:0.24; TC:0.1
2	1	8.69–10.24	T:0.21; TC:0.16; N:0.14; C:0.09
3	1	1.95–6.92	N:0.53; NY:0.12; IN:0.12; IY:0.05
4	1	0.9–6.51	TC:0.93
5	1	7.91–9.24	T:0.74
6	1	1.8–7.59	T:0.88
7	1	0.87–4.52	R:0.51; RC:0.16; TR:0.09; TC:0.06
8	1	0.55–3.09	RC:0.58; TRC:0.19; ERC:0.05
9	0.91	0.37–2.25	TBERC:0.23; TERC:0.19T; BRC:0.13; TRC:0.12; BERC:0.09
10	1	0.16–3.37	T:1
11	0.92	2.47–8.35	T:0.66; I:0.09; TB:0.05
12	0.91	0.9–6.53	T:0.83; TB:0.08; B:0.07
13	1	0.42–3.89	TB:0.5; B:0.28; T:0.22
14	0.98	0.19–2.15	TB:0.95
15	1	0–0.72	TB:0.99
16	0.99	0.14–3.26	T:1
17	1	0–0.81	INY:1

Bayesian posterior probabilities (BPP > 0.9), 95% highest posterior density (HPD) intervals (millions of years = Ma) based on a relaxed molecular-clock analysis of ITS-ETS sequences in BEAST. Letters correspond to the following ancestral areas or combination of areas with a relative probability (BayArea + J model) equal or greater 0.05. *A* Alps, *B* Balkans, *C* Transcaucasus, *E* Eurasia, *I* Iberian Peninsula, *N* North Africa, *R* Iran, *T* Turkey, *Y* south Italy.

The six 6 different BEAST models were compared according to their respective log values of Marginal Likelihood Estimators (MLE) that were obtained with path-sampling (PS) and stepping-stone (SS)^85^ implemented in BEAST v.1.8.4 (see Table S1). PS and SS log values were estimated with 100 path steps, a chain length of 106 generations and likelihoods saved every 1000 generations. The resulting log values of MLEs were averaged across four replicate runs to generate a single PS and SS value for each model. The obtained averages of the log values of MLEs for all hypotheses were ranked, and Bayes factors (BF) were then calculated using the modification introduced by^86^ (i.e., twice the difference between the harmonic mean likelihoods of the two models). Values for 2BF those are greater than 2, 6, and 10 indicate positive, strong, and decisive support, respectively, for the generic hypothesis with minor marginal likelihood.

The best model with decisive support was that with a relaxed clock with exponential distribution and Birth and Death speciation process (Table S1). After discarding the burn-in steps (25%), tree files from the four independent runs of the selected model were combined using LogCombiner 1.8.4 and the resulting maximum clade credibility (MCC) tree was summarized in TreeAnnotator 1.8.4 (https://beast.community/2016-06-17_BEAST_v1.8.4_released.html) and viewed in FigTree v.1.4.2 (https://github.com/rambaut/figtree/releases/tag/v1.4.4.).

### Ancestral area estimation

We inferred the origin of the genus *Rhaponticoides* and its possible routes of expansion and speciation from the interspecific phylogenetic relationships supported by a time-calibrated tree that was subjected to a biogeographical analysis, BioGeoBEARS^87^. We defined 9 geographic regions based, mainly, on the richness and endemicity of species in *Rhaponticoides* genus (Fig. 4, above). The time-calibrated tree resulting from Bayesian inference of BEAST was used as an input file to estimate the probabilities of ancestral ranges (dataset 3). BioGeoBEARS calculates maximum-likelihood estimates of the ancestral states at internal nodes by modeling transitions between geographical ranges along phylogenetic branches as a function of time. BioGeoBEARS encompasses six different biogeographical models (DEC, DEC + J, DIVA, DIVA + J, BAYAREA-LIKE, BAYAREA-LIKE + J) as implemented in the R package BioGeoBEARS^87^. All models entail dispersal (d) and extinction (e) as free parameters. Three models also comprise an additional parameter “j” (+ J) to embrace the founder-event speciation^87^ but DEC + J was discarded according to recent criticisms^88^. BioGeoBEARS yields maximum-likelihood estimates of the ancestral states at internal nodes by modeling transitions between geographical ranges along phylogenetic branches as a function of time. The fit of the six biogeographical models was compared using likelihood values and the Akaike Information Criterion (AIC). Thus, in our study, the BayArea + J model performed best (Table S2).

### Ethical statment

All samples included in the study come from specimens sampled with the respective permits from national administrations and herbaria (ATHU, National and Kapodistrian University of Athens, Greece; KNYA Konya Herbarium, Turkey; BC, Institut Botànic de Barcelona, Spain; CIEF, Servicio de Vida Silvestre, Generalitat Valenciana, Spain; E, Royal Botanic Garden Edinburgh, Scotland; HLUC, Università degli Studi della Basilicata, Italy; HYE, Conservatoire botanique national méditerranéen de Porquerolles, France; GAZI, Gazi University, Turkey; SEV, Universidad de Sevilla, Spain; SOM, Herbarium of the Institute of Biodiversity and Ecosystem Research, Bulgarian Academy of Sciences, Bulgary).

## Supplementary Information

Supplementary Tables.

## Data Availability

Datasets 1, 2, and 3 are accessible online at https://www.ibb.csic.es/public/. Vouchers specimen data and GenBank accession numbers of sequences are described in Table 1.
