# Supplementary material for: Biogeography of Rhaponticoides, an Irano-Turanian element in the Mediterranean flora
Source: Sci Rep. 2022 Dec 20;12:22019. doi: 10.1038/s41598-022-24947-3 (PMC9768164; doi:10.1038/s41598-022-24947-3)
Supplement: Supplementary file 1 — Supplementary Tables. [file 41598_2022_24947_MOESM1_ESM.docx]

**Supplementary information:**

**Tables S1, S2, and S3.**

**Table S1.** Bayes Factor (BF) comparisons for selection of substitution model and partitioning Scheme using combined nuclear data. PS = Path sampling log marginal likelihood; SS= Sttepping-stone log marginal likelihood. We consider 2BF > 10 as definitive evidence for a hypothesis (Kass and Raftery 1995). Asterisk * indicates the optimal model type and its PS and SS values.

| **Clock / Distribution / Speciation process** | **PS** | **2BF** | **SS** | **2BF** |
| --- | --- | --- | --- | --- |
| Relaxed / Exponencial / Birth & Death* | -4443.50* |  | -4435.92* |  |
| Relaxed / Exponencial / Yules | -4450.49 | 13.97 | -4450.20 | 13.39 |
| Relaxed / LogNormal / Birth & Death | -4450.76 | 14.52 | -4450.63 | 14.25 |
| Relaxed / LogNormal / Yules | -4451.83 | 16.66 | -4451.59 | 16.18 |
| Strict Clock / - / Birth & Death | -4865.31 | 843.60 | -4734.52 | 582.03 |
| Strict Clock / - / Yule | -4902.15 | 917.29 | -4815.93 | 744.85 |

**Table S2.** Akaike information criteria (AIC), natural log of the likelihood (LnL), number of parameters, dispersal, extinction, and founder effect values from six different biogeographical models. Asterisk * indicates the optimal model.

| **MODEL** | **AIC** | **LnL** | **Nº parameters** | **d** | **e** | **j** |
| --- | --- | --- | --- | --- | --- | --- |
| DEC | 304.1878 | -150.0939 | 2 | 0.0447 | 0.0903 |  |
| DIVA | 316.7592 | -156.3796 | 2 | 0.0802 | 0.2639 |  |
| DIVA+J | 319.7723 | - 156.8862 | 3 | 0.0432 | 0.0318 | 0.0152 |
| BAYAREA | 233.5950 | - 114.7975 | 2 | 0.0243 | 0.2534 |  |
| BAYAREA+J* | 211.7694* | -102.8847 | 3 | 0.0142 | 0.0336 | 0.0432 |

**Table S3. Summary of nucleotide variation obtained using *DnaSP* software (DNA Sequence Polymorphism, http://www.ub.edu/dnasp/).**

| **ADN region** | **Nº taxa** | **Nº nucleotides** | **Nº Polymorphic sites / mutations** | **Parsimony informative sites** |
| --- | --- | --- | --- | --- |
| ETS | 20 | 1027 | 77 / 80 | 66 |
| ITS | 20 | 567 | 28 / 31 | 19 |
| rpl32-trnL^UAG^ | 20 | 967 | 21 / 22 | 20 |
| ycf3-trnS | 20 | 854 | 22 / 22 | 21 |
